# Triclosan Demonstrates Synergic Effect with Amphotericin B and Fluconazole and Induces Apoptosis-Like Cell Death in *Cryptococcus neoformans*

**DOI:** 10.3389/fmicb.2016.00360

**Published:** 2016-03-21

**Authors:** Elaheh Movahed, Grace Min Yi Tan, Komathy Munusamy, Tee Cian Yeow, Sun Tee Tay, Won Fen Wong, Chung Yeng Looi

**Affiliations:** ^1^Department of Medical Microbiology, Tropical Infectious Disease Research and Education Center, Faculty of Medicine, University of MalayaKuala Lumpur, Malaysia; ^2^Department of Pharmacology, Faculty of Medicine, University of MalayaKuala Lumpur, Malaysia

**Keywords:** antifungal effect, amphotericin B, apoptosis, *Cryptococcus neoformans*, fluconazole, synergy, triclosan

## Abstract

**Objectives:** *Cryptococcus neoformans* is an opportunistic fungus that causes fatal meningoencephalitis especially in AIDS patients. There is an increasing need for discovery of new anti-cryptococcal drugs due to emergence of resistance cases in recent years. In this study, we aim to elucidate the antifungal effect of triclosan against *C. neoformans*.

**Methods:** Minimal inhibitory concentration (MIC) of triclosan in different *C. neoformans* strains was first examined. The *in vitro* interactions between triclosan and two standard anti-fungal drugs (amphotericin B and fluconazole) were further evaluated by microdilution checkerboard assay. Mechanism of triclosan fungicidal activity was then investigated by viewing the cell morphology under transmission electron microscope.

**Results:** We reported that triclosan potently inhibited the growth of *C. neoformans*. A combination of triclosan with amphotericin B or with fluconazole enhanced their fungicidal effects. Triclosan-treated *C. neoformans* displayed characteristics such as nuclear chromatin condensation, extensive intracellular vacuolation and mitochondrial swelling, indicating that triclosan triggered apoptosis-like cell death.

**Conclusion:** In summary, our report suggests triclosan as an independent drug or synergent for *C. neoformans* treatment.

## Introduction

Infection by *Cryptococcus neoformans* has become a major cause of mortality following the increased numbers of AIDS patients (Mitchell and Perfect, 1995; Pancharoen et al., 2001) and is estimated to cause approximately 600,000 deaths worldwide annually (Park et al., 2009). *C. neoformans* exists ubiquitously in the environment, commonly found in decaying wood and bird excreta (Lazera et al., 1996). Most individuals are exposed to *C. neoformans* through inhalation from the environment during their childhood but stay asymptomatic (Goldman et al., 2001) because *C. neoformans* is an opportunistic pathogen which rarely progresses to disease in immune competent individuals. However, it can result in life-threatening infection in immunosuppressed or immunocompromised patients (Coelho et al., 2014). Infection of *C.* *neoformans* in lung leads to cryptococcal pneumonia whereas systemic dissemination often causes fatal meningoencephalitis (Lin and Heitman, 2006).

In recent years, the widespread use of anti-microbial drugs in the treatment of fungal infection has led to the global emergence of resistant fungal strains. Numerous fungal pathogens including *C. neoformans* have demonstrated increasing resistance to common antifungal drugs such as amphotericin B and fluconazole (Perfect and Cox, 1999). A recent global study of nearly three thousand *C. neoformans* isolates shows that >11% of the isolates are resistant to fluconazole (Pfaller et al., 2009). Fluconazole-heteroresistant phenotype of *C. neoformans* has also been detected in a significant proportion of clinical isolates (Yamazumi et al., 2003). Increased drug resistance in fungal species is mainly attributed to overexpression and hotspot mutations of genes that encode for efflux ATP-binding cassette (ABC) transporters (Sanglard et al., 1995; Looi et al., 2005) such as CDR1, CDR2, and MDR1 (Fling et al., 1991; Prasad et al., 1995; Sanglard et al., 1997). Changes in fungal cell phospholipids and membrane sterol composition can also reduce drug permeation ability (Hitchcock et al., 1986). Alternate usage of other enzymes in the same biosynthetic pathway as a substitute for the drug-targeted enzyme also confers resilience and endurance to the yeasts (Howell et al., 1990). Therefore, studies to discover new drugs or combination of multiple anti-microbial drugs are essential. Some drug combinations demonstrate synergistic effect and superiority to the currently available therapies (Mukherjee et al., 2005; Munoz et al., 2006). For instance, combinational usage of fluconazole plus amphotericin B adds benefit in candidemia treatment (Odds, 2003a).

Triclosan (2,4,4′-trichloro-2′-hydroxydiphenylether, C_12_H_17_ Cl_3_O_2_) is a chlorinated compound which is widely used for personal care products such as soap, toothpaste and plastics in domestic as well as healthcare settings due to its safety, efficiency and long-lasting effects (Jones et al., 2000). Importantly, various studies using different test systems indicate that triclosan is a non-mutagenic and non-genotoxic agent (Fang et al., 2010). The drug is able to persist for at least 0.7 h (Loftsson et al., 1999) in human fluids, including nasal secretion, serum, urine, and milk after exposure (Syed et al., 2014). Triclosan demonstrates broad-spectrum anti-microbial properties against various species of microorganisms with MICs ranging from 0.1 to 30 mg/l (Schweizer, 2001) including *Candida* species (Vischer and Regos, 1974; Regos et al., 1979). Previous studies suggest that triclosan likely perturbs cell structure and results in loss of permeability-barrier function (Villalain et al., 2001). It inhibits bacterial and fungal fatty acid synthetic enzyme, by targeting *Fab1*–encoding NADH-dependent enoyl-acyl-carrier protein reductase (McMurry et al., 1998; Heath et al., 1999; Stewart et al., 1999).

Although the antifungal effect of triclosan has been reported in *Candida albicans* and several fungal species (Vischer and Regos, 1974; Regos et al., 1979; Yu et al., 2011), at present, the effect of triclosan in *C. neoformans* has not been investigated. In this study, the inhibitory effect of triclosan as well as the fungicidal synergism between triclosan and standard antifungal drugs against *C. neoformans* were evaluated. In addition, we examined the mechanism of antifungal action by viewing triclosan-treated cells under electron microscopy.

## Materials and Methods

### Fungal Isolates and Chemicals

*Cryptococcus neoformans* H99, *C. albicans* 90028 and *C. albicans* SC5314 (MYA2876) were obtained from American Type Culture Collection (ATCC). *C. neoformans* C14 and C17 strains were isolated from inpatients at the University of Malaya Medical Center. *C. neoformans* H4, S48B, and S68B environmental strains were isolated from bird droppings at different locations in Klang valley, Malaysia (Tay et al., 2005). All *C. neoformans* strains used were *C. neoformans var. grubii*, the predominant serotype A, genotype VNI with an α-mating type (Tay et al., 2006).

Amphotericin B, fluconazole, and triclosan were purchased from Sigma–Aldrich (St. Louis, MO, USA). Stock solutions were prepared at 10 mg/ml by dissolving the amphotericin B and triclosan powder in Dimethyl sulfoxide (DMSO), and fluconazole in water. Drugs were stored at -20°C until use.

### Disk Diffusion Assay

Five single colonies were picked and inoculated into Sabouraud dextrose broth (SDB). Cells were grown overnight in a rotary shaker at 200 rpm at 35°C. An aliquot of 100 μl of the yeast suspension at 10^6^ CFU/ml was prepared, applied onto the Sabouraud dextrose agar (SDA) plate and spread uniformly using a cotton swab. Then, 6-mm paper disks impregnated with different concentrations of triclosan (3.125, 6.25, 12.5, 25, 50, and 100 μg) were positioned on a *C. neoformans* H99 agar plate to evaluate the antifungal effect of triclosan. In the synergy test, 25 μg of a drug alone or in combination were placed onto the agar surface. The synergy test was repeated using sub-inhibitory concentrations of 6.25 μg/ml from AMB and FLU alone and in combination with 6.25, 3.125, and 1.56 μg/ml of triclosan. Dimethyl sulfoxide (DMSO) was used as negative control. After incubation at 37°C for 48 h, the diameters of the growth inhibition zone were measured.

### Broth Microdilution Assay

The drug minimum inhibition concentration (MIC) was determined by broth microdilution assay according to Clinical and Laboratory Standards Institute (CLSI) standard (Clinical and Laboratory Standards Institute [CLSI], 2008). The inoculum was prepared by picking five colonies (∼1 mm diameter) from a fresh culture plate. Colonies were resuspended in 2 ml distilled water and vortexed for 15 s. The cell density was adjusted to 75% transmittance at 530 nm wavelength using a spectrophotometer. A working suspension was made by a 1:50 dilution followed by a 1:10 dilution of the stock suspension with RPMI 1640 medium supplemented with 34.53 mg/ml morpholinepropanesulfonic acid (MOPS) at pH 7.0 to yield yeast stock suspension of 1.0 to 5.0 × 10^3^ cells/ml. Antifungal drugs, amphotericin B (32 to 0.017 μg/ml), fluconazole (128 to 0.068 μg/ml), and triclosan (128 to 0.068 μg/ml) were serially diluted in 96-well flat-bottomed microtiter plates. The cells suspension (100 μl) were then seeded into the plate and incubated for 48 h. The microtiter plates for *C. albicans* and *C. neoformans* were visually scored after incubation at 37°C for 24 and 48 h, respectively (Tay et al., 2011). Each well was resuspended and agitated for 5 min, and the optical density (OD) at 570 nm wavelength was determined with a spectrophotometer. All samples were run in duplicate. The MIC-1 was defined as the minimal concentration that resulted in 80% growth inhibition while MIC-2 was defined as the minimal concentration that resulted in 50% of growth inhibition (Yu et al., 2011).

### Fungicidal Assay

The MFCs of triclosan was determined by conventional culture-based CFU method from microtitration plate in duplicates, as previously described (Meletiadis et al., 2007). Total amount of 20 and 100 μl from all visually clear wells and the first well with the highest drug concentration showing growth (0.5x MIC) were mixed by pipetting up and down several times, washed and subcultured onto SDA plates in duplicates. The SDA plates were incubated at 37°C for 48 h, and the CFU were observed for each drug concentration. The MFCs were defined as the lowest drug concentration yielding no growth using 20 μl (CFU20 MFC) and 100 μl (CFU100 MFC). Triclosan was considered fungicidal when the MFC/MIC ratio is ≤4 and fungistatic when the MFC/MIC ratio is ≥4 (Pfaller et al., 2004; Meletiadis et al., 2007).

### Synergy Checkerboard Assay

Antibiotic interactions were evaluated using the checkerboard assay as previously described (Rand et al., 1993). Checkerboards were prepared by using serial dilutions of amphotericin B (0.004 to 4.0 μg/ml) and fluconazole (0.0156 to 16.0 μg/ml) in the horizontal wells and triclosan (0.25 to 16.0 μg/ml) in the vertical wells. A fungal suspension was prepared as described in broth microdilution assay (approximately 5 × 10^3^CFU/ml), and 100 μl was inoculated into each well of a 96-well microtiter plate. Plates were read after 48 h of incubation at 35°C, and the wells without visible signs of growth were identified by placing the plate on a mirrored surface. Each well was resuspended and agitated for 5 min, and the OD at 570 nm wavelength was determined with a spectrophotometer. The fractional inhibitory concentration index (FICI) was calculated for each drug using following formula: FICI = FIC (triclosan) + FIC (drug), where FIC equals to MIC-2 of the drug in combination divided by the MIC-2 of the drug alone. FICI ≤ 0.5 indicates synergy, FICI > 4 indicates antagonism whereas 0.5 > FICI > 4 suggests no interaction (additivity/indifference; Odds, 2003b).

### Electron Microscopy

Yeast cells were pelleted by centrifugation and resuspended in 5 ml of distilled water. The turbidity of the suspension was adjusted with a spectrophotometer to 75% transmittance at 530 nm. The distilled suspension was diluted 1:50 (0.1 ml plus 4.9 ml) with RPMI 1640 medium. The cell suspension was treated with 0.5 μg/ml of triclosan at 37°C, for 2 h. Cells were then collected and fixed with 4% glutaraldehyde fixative for 120 min. Cells were washed few times with cacodylate buffer and post fixed with OsO_4_:cacodylate buffer (1:1) for 2 h and kept in cacodylate buffer at 4°C overnight. The samples were washed with distilled water, incubated with uranyl acetate for 10 min before another washing step. Dehydration was performed using 35, 50, 75, and 95% (v/v) ethanol for 10 min each, and followed by 100% ethanol, 15 min for three times. Samples were then incubated with propylene oxide:epon (1:1) for 1 h, propylene oxide:epon (1:3) for 2 h and epon for an overnight incubation. This was followed by embedding the yeast pellet in resin for 5 h at 37°C followed by 60°C overnight. Images were obtained using EFTEM LIBRA 120 transmission electron microscope (Carl Zeiss, Oberkochen, Germany).

### Apoptosis Assay

Cell apoptosis was examined by terminal deoxynucleotidyl transferase (TdT)-mediated dUTP nick end labeling (TUNEL) assay using FLOWTAC kit (Trevigen, Gaithersburg, MD, USA). Log-phase cultured cells (10^7^ cells) were treated with 2 mM hydrogen peroxide (H_2_O_2_) or 0.5 μg/ml triclosan for 4 h at 37°C. Cells were fixed in 1 ml 3.7% formaldehyde for 10 min followed by Cytonin^TM^ permeabilization for 30 min. Cells were then washed and resuspended in labeling reaction mix (TdT dNTP mix, TdT enzyme, 1x Mn^2+^, 1x TdT labeling buffer) at 37°C for 1 h. Reaction was stopped by adding 1x Stop Buffer followed by staining with 25 μl Strep-Fluorescein for 10 min in dark. Propidium iodide and RNase were added to the cells before analyzed by a FACS Canto cytometer (BD Biosciences, Franklin Lakes, NJ, USA).

### Statistical Analysis

Data were analyzed with unpaired two-tailed Student’s *t*-test. Samples were considered significant if *P* < 0.05.

## Results

### Triclosan Inhibits the Growth of *C. neoformans*

**FIGURE 1:**
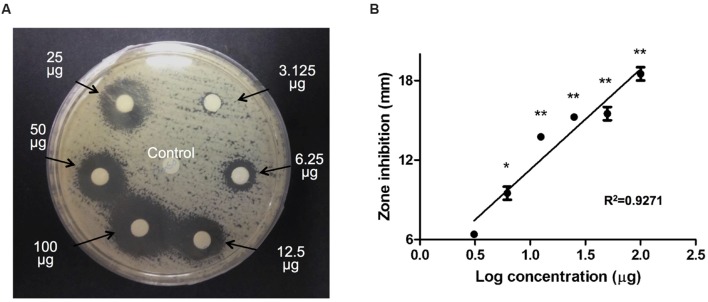
**Agar disk diffusion assay.** **(A)** Paper disks impregnated with control DMSO or 3.125, 6.25, 12.5, 25, 50, and 100 μg of triclosan were placed on the agar plate containing *Cryptococcus neoformans*. Diameters of the inhibition zone were measured after 48 h of incubation. Data shown is a representative picture of three independent experiments. **(B)** Graph shows the inhibition diameter (mm) of *C. neoformans* plate after triclosan treatment. Data shown are mean ± SD. ^∗^*P* < 0.05, ^∗∗^*P* < 0.01.

To evaluate the antifungal effect of triclosan, 6-mm paper disks impregnated with different concentrations of triclosan (3.125, 6.25, 12.5, 25, 50, and 100 μg) were positioned on a *C. neoformans* H99 agar plate for an incubation period of 48 h. Control disk (with DMSO) displayed no inhibitory zone. In contrast, we noted that the diameters of the inhibition zones surrounding the triclosan-impregnated disks increased steadily from 7 to 17.6 mm in a dose-dependent manner (**Figure 1A**). A linear relationship was observed between the inhibition zone size and Log concentration of triclosan (**Figure 1B**).

Triclosan MIC-1 and MIC-2 endpoint values, which represent reduction of cell turbidity at 80 and 50%, respectively, were measured by broth microdilution assay (**Table 1**). Triclosan was fungicidal against *C. neoformans* H99, with MIC-1 = 3.80 and MIC-2 = 2.70 μg/ml. Local clinical isolates of *C. neoformans* tested (C14 and C17) showed MIC-1 at 5.197 and 4.875 μg/ml, and MIC-2 at 2.130 and 2.280 μg/ml. Environmental strains *C. neoformans* H4, S48B and S68B strains showed comparable MIC-1 and MIC-2 values ranging from 0.25 to 1.14 μg/ml and 0.08 to 0.54 μg/ml, respectively (**Table 1**). Further, we showed that the MIC-1 and MIC-2 of triclosan in two *C. albicans* (90028 and SC5314) strains were between 16 and 64 μg/ml, consistent with a previous report (Yu et al., 2011). The relatively lower MIC values in *C. neoformans* suggest that the triclosan is more potent against *C. neoformans* compared to *C. albicans.*

**Table 1:** Minimal inhibitory concentration-1 (80% inhibition) and MIC-2 (50% inhibition) readings of triclosan in different strains of *Cryptococcus neoformans* and *Candida albicans*.

Fungal strains	MIC-1 (μg/ml)	MIC-2 (μg/ml)
*Cryptococcus neoformans H99*	3.80	2.70
*Cryptococcus neoformans C14*	5.197	2.130
*Cryptococcus neoformans C17*	4.875	2.280
*Cryptococcus neoformans H4*	1.14	0.54
*Cryptococcus neoformans S48B*	0.37	0.12
*Cryptococcus neoformans S68B*	0.25	0.08
*Candida albicans 90028*	59.6	44.0
*Candida albicans SC5314*	>64	33.8


*Data are representative of three independent experiments.*

A cell culture CFU-based fungicidal assay was then performed to determine if the triclosan was fungicidal or fungistatic (**Table 2**). CFU formation can be visualized in the wells with low concentrations (≤0.5 μg/ml) but no CFU was detected in the media recovered from the wells treated with ≥1 μg/ml, indicating that MFC for *C. neoformans* H99 was 1 μg/ml. Thus, our data suggest that triclosan wasfungicidal (MFC/MIC-2 ratio = 0.37, ≤4).

**Table 2:** CFU count.

Triclosan (μg/ml)	CFU count
0.065	>300
0.125	177
0.25	163
0.5	96
1	0
2	0


*Total CFU count in the cell culture CFU-based fungicidal assay. Data shown are representative of two independent experiments.*

### Triclosan Demonstrates a Synergistic Effect with Amphotericin B and Fluconazole

**FIGURE 2:**
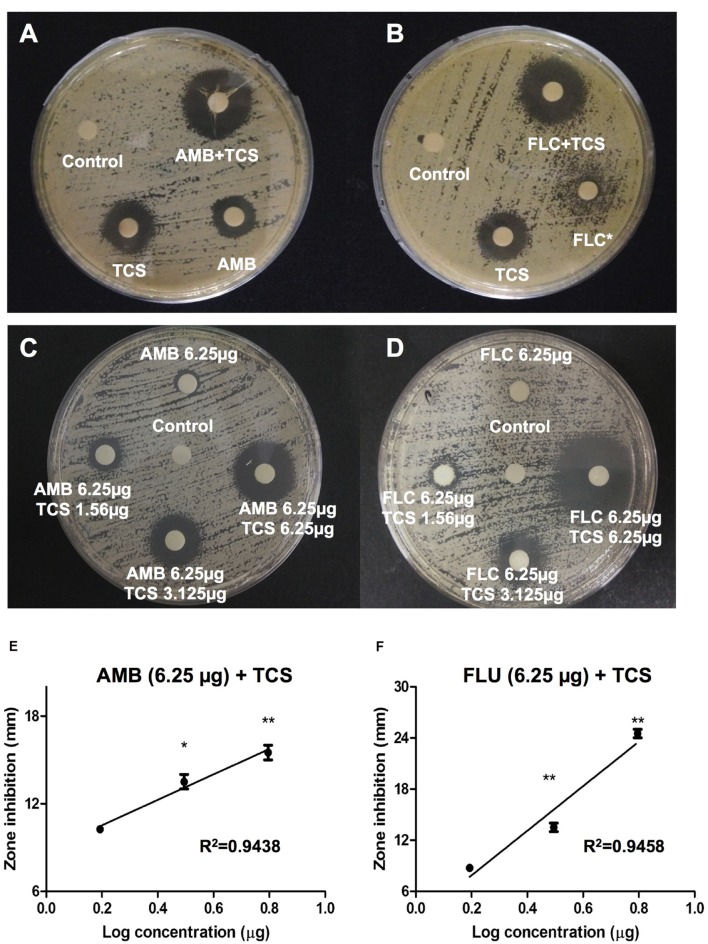
**The effect of triclosan with amphotericin B and fluconazole.** **(A,B)** Agar disk diffusion assay for triclosan (TCS) in combination with **(A)** amphotericin B (AMB), or **(B)** fluconazole (FLC) in *C. neoformans* H99-containing agar plates. 25 μg of each drug was applied on a disk. **(C,D)** Agar disk diffusion assay for TCS (1.56, 3.125, and 6.25 μg/ml) in combination with at subinhibitory concentrations (6.25 μg/ml) of **(C)** AMB and **(D)** FLU. Diameters of the inhibition zone were measured after 48 h of incubation. Asterisk ^∗^ indicates partial inhibition. Data shown are representative pictures of three independent experiments. **(E,F)** Graphs show the inhibition diameter (mm) of *C. neoformans* plate after triclosan treatment in combination with AMB or FLU. X-axis shows Log concentration of triclosan. Data shown are mean ± SD. ^∗^*P* < 0.05, ^∗∗^*P* < 0.01.

*In vitro* interactions between triclosan and two standard antifungal agents, amphotericin B and fluconazole were examined using a disk diffusion assay (**Figure 2**). Inhibition zone for amphotericin B treatment alone was 10 mm. Noticeably, combinational usage of triclosan plus amphotericin B caused enlargement of the inhibition zone to 17 mm (**Figure 2A**). On the other hand, fluconazole treatment alone showed incomplete inhibition zone of approximately 11 mm, while the combinational usage of triclosan plus fluconazole remarkably augmented the size of inhibition zone to 16 mm (**Figure 2B**).

To further confirm the result, we repeated the experiment using subinhibitory concentrations of drugs. Increasing concentrations of triclosan at 1.56, 3.125, and 6.25 μg/ml were applied on the paper disks containing 6.25 μg amphotericin B (**Figure 2C**) or fluconazole (**Figure 2D**) and used for disk diffusion assay. No or minimal inhibition zones were observed in the absence of triclosan while addition of triclosan caused enlargement of inhibition zone in a dose-dependent manner. Both combinations of triclosan with amphotericin B or fluconazole showed linear inhibition patterns (**Figures 2E,F**). These results suggest that the effect of triclosan with the standard drugs is either synergistic or additive.

The combinational effect of standard drugs and triclosan was further assessed using checkerboard assay to define the median FICI values (**Table 3**). Both combination of drugs, i.e., triclosan plus amphotericin B (FICI = 0.127), or triclosan plus fluconazole (FICI = 0.020) showed a FICI value <0.5, indicating the synergistic (but not additive) effect of triclosan with both standard drugs.

**Table 3:** Checkerboard assay.

	MIC alone	MIC combined	FIC	FICI	Note
Amphotericin B	0.23	0.008	0.035	0.127	Synergy
Triclosan	2.7	0.25	0.092		
Fluconazole	3.2	0.015	0.005	0.020	Synergy
Triclosan	2.7	0.06	0.022		


*Synergy effect of triclosan with amphotericin B or fluconazole in *C. neoformans*. FICI ≤ 0.5 indicates synergistic effect. Data shown are representative of two independent experiments.*

### Triclosan Triggers Apoptotic-Like Cell Death in *C. neoformans*

**FIGURE 3:**
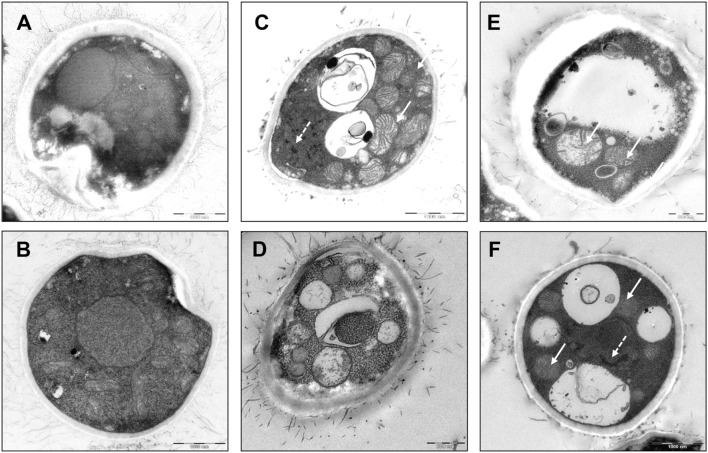
**Cell morphology of triclosan-treated *C. neoformans*.** Electron microscopic pictures of *C. neoformans* with or without exposure to triclosan (0.5 μg/ml) for 2 h. **(A,B)** Untreated control cells showed intact nucleus and cytoplasm. **(C–F)** Triclosan-treated *C. neoformans* showed apoptotic morphologies. Note the appearance of apoptotic features such as mitochondrial swelling (arrow) and nuclear chromatin condensation (broken arrow). Besides, intense cytoplasmic vacuolations were formed in the cells. Cell surface fibrilar structures which form capsule polysaccharide component were also disrupted (Pavel and Vasile, 2012).

To examine the inhibitory mechanism of triclosan on *C. neoformans*, we processed the control and triclosan-treated (2 h) cells for visualization under electron microscope. Untreated *C. neoformans* showed an intact cell structure with normal morphologies of nucleus and cytoplasm (**Figures 3A,B**). In contrast, triclosan-treated *C. neoformans* demonstrated disrupted cell morphologies including apparent mitochondrial swelling and extensively enlarged cytoplasmic vacuolations (**Figures 3C–F**). Nuclear chromatin condensation can be also observed. The cell surface fibrilar structure which constitutes polysaccharide component of the cell wall capsule was also disrupted (Pavel and Vasile, 2012). These features collectively suggest that triclosan-treated *C. neoformans* demonstrated apoptotic-like cell death (ALCD) mechanism.

**FIGURE 4:**
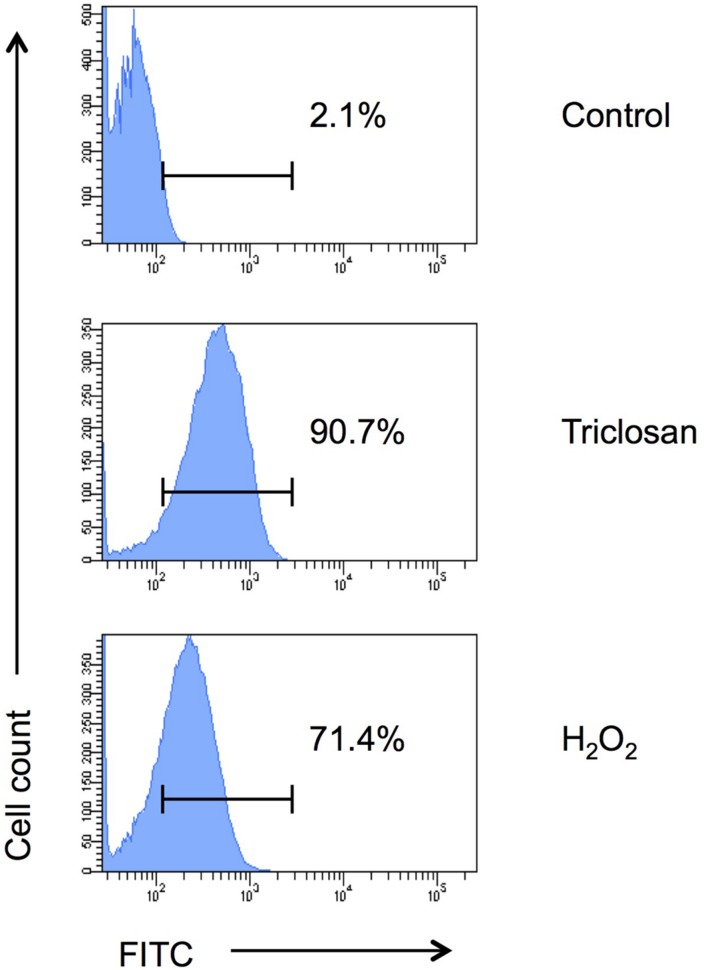
**TUNEL assay.** Apoptosis of triclosan-treated *C. neoformans* H99 cells was determined by TUNEL assay followed by flow cytometry analysis. *C. neoformans* H99 cells were non-treated (control), treated with triclosan (0.5 μg/ml) or H_2_O_2_ (2 mM) for 4 h. Percentages of the apoptotic cells were indicated. Data shown are representative of two independent experiments.

To further confirm if triclosan triggers apoptosis in *C. neoformans* cells, we analyzed the cells using TUNEL apoptosis assay followed by flow cytometrical analysis (**Figure 4**). DNA fragmentation (one of the hallmark of apoptosis) generates free 3′-hydroxyl residues that can be utilized by terminal deoxynucleotidyl transferase to incorporate FITC-tagged dUTP into the blunt ends of double-stranded DNA break. Our data showed that 90.7% triclosan-treated *C. neoformans* H99 cells were apoptotic (FITC-positive) compared to only 2.1% in the untreated control. Around 71.4% of apoptotic cells were detected in the positive control (H_2_O_2_-treated cells). Therefore, triclosan can inhibit *C. neoformans* by inducing ALCD.

## Discussion

Our study demonstrated the potent antifungal effect of triclosan against *C. neoformans*. In fact, triclosan treatment exerted a stronger growth inhibition in *C. neoformans* compared to *C. albicans*, as evidenced by comparatively lower MIC-1 values (ranged from 0.25 to 3.80 μg/ml) in *C. neoformans* strains tested, compared to MIC-1 values in *C. albicans* (ranged from 59.6 to >64 μg/ml).

Combined antibiotic therapy can delay the emergence of microbial resistance by producing desirable synergistic effects in the infection treatment (Adwan and Mhanna, 2008). In our study, synergism between triclosan and two standard drugs (amphotericin B and fluconazole) was established. Fluconazole and amphotericin B are commonly used drugs for candidiasis and cryptococcal diseases. Fluconazole is a member of the azole family that targets the Erg11 enzyme (an essential fungal cytochrome P450 lanosterol 14α-demethylase), thus inhibiting ergosterol biosynthesis (Rodero et al., 2003; Heilmann et al., 2010). Amphotericin B, on the other hand, binds directly to ergosterol and kills yeast cells via ion channel-mediated plasma membrane permeabilization (Gruda and Dussault, 1988). Combinational treatment of triclosan and other drugs has been shown to significantly enhance the drug efficacy against bacteria (Tambe et al., 2001; Sharma et al., 2003). However, two previous reports using *C. albicans* demonstrated opposing results for combinational usage of triclosan plus fluconazole, whereby one showed synergistic effect (Yu et al., 2011) and another showed antagonistic effect (Higgins et al., 2012). In *C. neoformans*, we reported that triclosan acts in synergy with fluconazole and amphotericin B.

Administration of standard fungal drugs in AIDS-associated cryptococcal meningoencephalitis shows a successful rate of 34% (fluconazole) in 40% in fluconazole and amphotericin B recipients (Saag et al., 1992). Furthermore, amphotericin B treatment frequently results in adverse effect (nephrotoxicity) in cryptococcal meningitis patients (Perfect et al., 2010), which suggests the need for a more effective treatment strategy. Perhaps a combinational therapy with triclosan could be a good solution. As far as safety is concerned, triclosan is considered non-carcinogenic with low indication of toxicity in both animal and human studies (Bhargava and Leonard, 1996). Triclosan has been impregnated to many of the daily contact products including soaps, toothpastes, disinfection solutions, and medical devices. Upon usage, triclosan can penetrate through cutaneous layer into the blood stream and excreted in the urine or feces (Moss et al., 2000; Sandborgh-Englund et al., 2006). Wound closure with triclosan-coated sutures has been shown to reduce the risk of infection after cerebrospinal fluid surgery (Rozzelle et al., 2008). Triclosan administration also significantly reduced bacterial level in experimental gingivitis (Pancer et al., 2016) and nosocomial infection (Webster et al., 1994).

The mechanism of triclosan-mediated cell disruption has been described in different pathogens. Triclosan triggers changes in bacteria membrane fluidity and function at lower concentration, and leads to cell lysis at a higher concentration (Gomez Escalada et al., 2005). It is also known to block lipid synthesis primarily by targeting the carrier protein of the bacterial type II fatty acid synthesis (FASII) pathway (McMurry et al., 1998). Contradictory, a recent study shows that *Fas1* and *Fas2* gene overexpression did not alter fungal susceptibility to triclosan and suggests that there may be an alternative target of triclosan in addition to the lipid synthesis pathway (Higgins et al., 2012). Therefore, further investigations are required to elucidate the actual mechanism of action. In mammals, triclosan antagonizes estrogen or androgen receptors, elevates resting cytosolic Ca^2+^ in primary skeletal myotubules (Ahn et al., 2008) and impairs the excitation contraction coupling of cardiac and skeletal function (Cherednichenko et al., 2012).

From electron microscope pictures, we hypothesize that triclosan induces ALCD, a program cell death mechanism in *C. neoformans* because various apoptosic subcellular changes can be visualized as early as 2 h post treatment. At an early stage of an apoptotic cell, cell shrinkage occurs as a result of organelle condensation and decreased cytoplasm density (Häcker, 2000). During the apoptosis process, free radicals can modify mitochondrial membrane potential thus inducing mitochondrial swelling and fusion of adjacent mitochondria into megamitochondria (Wakabayashi, 1999). Mitochondria release cytochrome c from storage which subsequently activates the caspase cascade leading to nuclear cleavage (Zhang and Xu, 2000) and extensive plasma membrane blebbing (Coleman et al., 2001). We reported some apoptotic features in triclosan-treated *C. neoformans* cells including condensed nuclear chromatin, DNA fragmentation and mitochondrial swelling. Whether the H_2_O_2_ treatment induces similar pattern of apoptotic features in *C. neoformans* remains to be investigated. We suspected that the intensive cytoplasmic vacuolation was due to the decreased cytoplasm density and organelles condensation although the cell structure remained intact as a resultant of rigid fungal cell wall. Several pathogenic fungi have been reported to undergo ALCD (Buttner et al., 2006; Ramsdale, 2008; Sharon et al., 2009). For instance, mutation of CDC48 in *Saccharomyces cerevisiae* shows apoptosis characteristic including chromatin condensation and fragmentation (Madeo et al., 1997). Interestingly, overexpression of mammalian anti-apoptotic Bcl2 rescues fungal cell apoptosis (Longo et al., 1997) while overexpression of pro-apoptotic Bax enhances the cell death effect (Manon et al., 1997; Ligr et al., 1998). ALCD has also been reported in *C. neoformans* when the cells were stimulated with hydrogen peroxide or cultured in the presence of *Staphylococcus aureus* (Ikeda and Sawamura, 2008). The apoptosis pathway in *C. neoformans* is controlled through regulation of apoptosis-inducing factor (Aif1) and metacaspases (Mca1 and Mca2) (Semighini et al., 2011).

TUNEL assay takes advantage of *in situ* labeling technology, which uses TdT to incorporate FITC-tagged dUTP into blunt ends of double stranded DNA breaks (Denton and Kumar, 2015). TUNEL-labeled cells indicate nuclear DNA fragmentation, an irreversible step in apoptosis (Lawry, 2004). During infection, *C. neoformans* is able to cause apoptotic cell death of mammalian cells. In the experimental animals with disseminated cryptococcosis, a major constituent of *C. neoformans* capsular polysaccharide, glucuronoxylomannan (GXM) results in increased TUNEL-labeled cells in both lung and spleen (Chiapello et al., 2003). Studies also show that *C. neoformans* induces cell death in rat macrophages by promoting inducible nitric oxide synthase expression with nitric oxide production in rat macrophages (Chiapello et al., 2008; Villena et al., 2008) besides interrupting the lysosome and phagosome maturation (Davis et al., 2015; Smith et al., 2015). In addition, *C. neoformans* induces apoptosis of T lymphocytes through activation of caspase-8 that initiates DNA cleavage activity (Pericolini et al., 2006).

In summary, our study reveals a strong inhibitory effect of triclosan as well as its synergy with amphotericin B and fluconazole in blocking cell growth of the pathogenic fungus *C. neoformans*. We also report that triclosan maybe fungicidal by inducing the ALCD mechanism in *C. neoformans*. Our study reveals a potential therapeutic value of triclosan as a novel drug or a synergent in the treatment of cryptococcal infection.

## Author Contributions

CYL, STT, and WFW designed the research and analyzed the data. EM, GMYT, KM, and TCY carried out the experiments. EM and WFW wrote the manuscript. All authors have read and approved the final manuscript.

## Conflict of Interest Statement

The authors declare that the research was conducted in the absence of any commercial or financial relationships that could be construed as a potential conflict of interest.
